# Wafer-scale radio frequency ZnO Schottky diodes and arithmetic circuits

**DOI:** 10.1038/s41598-025-06506-8

**Published:** 2025-07-08

**Authors:** Harold F. Mazo-Mantilla, Zhanibek Bizak, Linqu Luo, Hendrik Faber, Camelia Florica, Suman Mandal, Atif Shamim, Khaled N. Salama, Thomas D. Anthopoulos

**Affiliations:** 1 Materials Science and Engineering Department, Physical Science and Engineering (PSE) Division, King Abdullah University of Science and Technology (KAUST), KAUST Solar Center, Thuwal, 23955 Makkah, Saudi Arabia; 2 Electrical and Computer Engineering Department, Computer, Electrical and Mathematical Sciences and Engineering (CEMSE) Division, King Abdullah University of Science and Technology (KAUST), Thuwal, 23955 Makkah, Saudi Arabia; 3 Nanofabrication Core Lab (NCL), King Abdullah University of Science and Technology (KAUST), KAUST Core Labs, Thuwal, 23955 Makkah, Saudi Arabia; 4 Department of Electrical and Electronic Engineering, Henry Royce Institute, Photon Science Institute, The University of Manchester, Manchester, M13 9PL UK

**Keywords:** Nanoscale devices, Techniques and instrumentation, Electrical and electronic engineering

## Abstract

Modern telecommunication technologies, such as the 5G and upcoming 6G networks, rely on devices operating in the radio frequency (RF) spectrum of 0.3–90 GHz and 7–300 GHz, respectively. To meet these demanding frequency requirements, new manufacturing methods and device architectures are gaining increasing attention. However, achieving scalable manufacturing alongside ultra-fast device operation presents formidable techno-economic challenges. Here, we explored a modified version of adhesion lithography (a-Lith) to create coplanar nanogap zinc oxide (ZnO) Schottky diodes for application in diode-logic arithmetic circuits. The planar ZnO diodes offer highly scalable manufacturing and combine high current rectification (> 10^6^) with low reverse currents (≈80 pA) and a remarkable cut-off frequency of over 25 GHz. Engineering the topologies of the planar ZnO diodes enables their facile monolithic integration into multi-bit AND and OR gates over 4-inch glass wafers. By integrating several such logic gates, we demonstrated fully functional monolithic 2-bit Half-Adder circuits, the primary component of an arithmetic logic unit. The work offers an alternative method for developing fast large-area electronics that could lead to a new family of logic circuitry.

## Introduction

The modern integration of Internet of Things (IoT) platforms and fifth-generation (5G) networks calls for steady improvements in device performance and up-scalable fabrication processes^1–3^. In addition, the continuous technological growth sets a research path for the upcoming sixth-generation (6G) networks and relevant device ecosystem^4^. For applications in the 5G domain, the operational frequency spectrum spans from a few GHz to 71 GHz^5^, or even 100 GHz^6^, while for 6G, the range covers from 6 GHz up to 300 GHz^6,7^ for research activities^4,6^. The successful deployment of these technologies in an expanding range of applications relies on developing cheap devices with the required performance, such as efficient and high-frequency operation^8^. Important building blocks include diodes, transistors, radio frequency (RF) antennas^9,10^, energy conversion and storage elements^11^, electronic switches^12^, mixers^13^, multiplexors, and logic circuits^14^ among others.

A key building block of modern digital electronics is the Arithmetic Logic Unit (ALU), often considered the computational brain of any digital system. The ALU sequentially executes fundamental operations like comparison, addition, binary shifting, etc., to accomplish a variety of demanding computational tasks. While there is extensive work at the device level for ALUs^15^, advances in circuit architectures utilising diodes instead of transistors or other elements are limited. Although various families of diode-based circuits for logic and RF applications have been demonstrated, the manufacturing complexity and hence cost of those devices and circuits remains high while the often high temperature processing used impose limitations on the choice of substrate materials^16–18^. Considering the broad use of diodes in electronics and recent strides in device-level manufacturing and performance^1^, new diode logic circuits could be used to develop RF circuits, provided that the performance and manufacturability issues can be adequately addressed^16,18^.

The ideal diode designed for logic circuit applications should combine a high on/off current ratio (I_on/off_ > 10^4^) to allow for a clear distinction between the two binary states “1” and “0” in elementary logic circuits^19,20^. Integration of several of these logic blocks can then be implemented to form more complex circuitry. For example, integrating three AND with one OR gate allows replicating the function of a fundamental arithmetic logic device, namely the 2-bit Half-Adder^21^. Considering the two stages composing the Half-Adder circuit, the operating characteristics of the discrete diodes become critical in achieving reliable circuit operation^21,22^. Amongst the different technologies, Schottky diodes are promising candidates for implementation in logic circuits due to their advantageous operating characteristics, including low-voltage operation, fast switching and lower device complexity^17,23^. Crucially, Schottky diodes can be fabricated and integrated monolithically using various techniques over large-area substrates using industry-relevant processes^24^. A recently demonstrated process for coplanar Schottky diodes is adhesion lithography (a-Lith)^25^. The technique has been used to develop scalable planar nanogap electrode (NGE) Schottky diodes, making it ideal for application in large-area RF electronics^26,27^. Compared to the more traditional sandwich-type diode architectures, the planar layout of the a-Lith diodes allows for ultra-low capacitance and unprecedented cutoff frequencies^26,28^.

The semiconductor material used to construct the diodes also plays a crucial role. While different families of functional materials like organic semiconductors and two-dimensional (2D) materials have their advantages and disadvantages, metal oxide semiconductors are attractive due to their numerous attributes that include excellent charge transport, low temperature processing versatility, low material cost and mechanical compliance^27,29–31^. Zinc oxide (ZnO) is one of the most widely studied metal oxides and has been recently used to develop RF Schottky diodes^28,32^^,^^33^. Herein, we used a modified version of a-Lith to develop coplanar Al/ZnO/Au Schottky diodes and monolithically integrate them to form fully functional logic circuits. The as-prepared ZnO diodes exhibit over > 90% manufacturing yield, high current rectification (> 10^4^) and intrinsic cut-off frequencies in excess of 25 GHz. By monolithically integrating the ZnO diodes over 4-inch glass wafers we demonstrated complex diode-based logic circuits, such as AND and OR gates. Going a step further and integrating several diode-based logic gates enabled the demonstration of more complex 2-bit Half-Adder circuits, i.e. the primary component of any ALU. The proposed strategy offers an alternative approach to circuit development for the burgeoning sectors of 5G and 6G technologies.

## Results and discussion

**Fig. 1:**
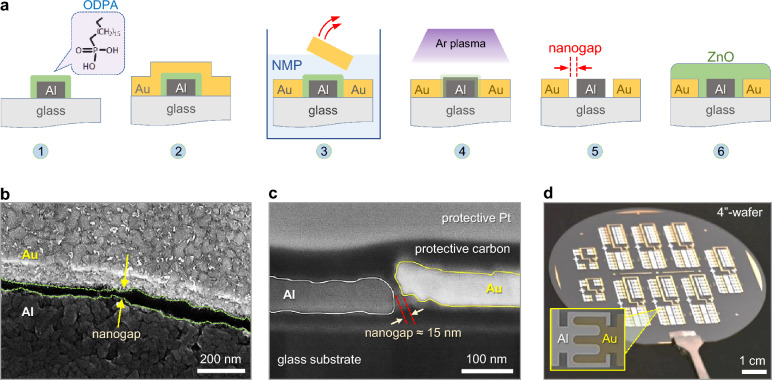
Fabrication and characterisation of asymmetric electrode nanogap Schottky diodes. **a**, Process steps used to fabricate the ZnO Schottky diodes based on Al and Au coplanar nanogap electrodes: (1) Deposition of the ODPA SAM on top of a patterned Al electrode, (2) blanket thermal evaporation of Au across the entire substrate, (3) nanogap development by removing the Au layer from areas where it overlaps with the Al/ODPA electrodes via dipping in hot NMP, (4) SAM removal by brief exposure to argon plasma, (5) the empty Al-Au nanogap electrodes, (6) ZnO deposition onto the nanogap electrodes via sequential spin coating and thermal annealing. **b**, SEM top-view image of a representative Al-Au nanogap. **c**, High-resolution TEM cross-sectional view of the Al-Au nanogap revealing a ≈15 nm inter-electrode distance. **d**, Photography of a 4-inch glass wafer containing NGE-based ZnO Schottky diodes and circuits. The inset shows a zoomed image of a discrete interdigitated NGE-based ZnO diode.

The fabrication steps used to develop the asymmetric coplanar nanogap electrodes of aluminum (Al) and gold (Au) are schematically depicted in Fig. 1a. In brief, an Al layer (metal-1, M1) is first deposited atop a glass wafer and patterned via standard photolithography. Following, a self-assembled monolayer (SAM) of octadecylphosphonic acid (ODPA) is applied onto the mildly oxidized surface of M1 (step-1). The deposition of Au as a second metal (metal-2, M2) produces a work function (Φ) difference with metal-1 (Al), which is vital to forming the planar Schottky diodes (step-2). The surface of the Al/ODPA electrode is highly hydrophobic due to the SAM’s methyl end groups^25^, drastically reducing the adhesion force with the subsequently deposited Au (M2), whereas the adhesion of M2 on bare glass substrate remains high. By immersing the as-prepared sample in N-Methyl-2-pyrrolidone (NMP) and sonicating it for several minutes, the regions of M2 overlapping with M1/SAM undergoes self-delamination, leaving behind laterally aligned Al/ODPA-Au electrodes (step-3). The ODPA was removed from the surface of M1 via exposure to argon plasma for 10 min (step-4) leaving behind a clean nanogap formed between M1 and M2 (step-5). Diode fabrication was completed with the deposition of ZnO via spin coating followed by thermal annealing (step-6) in air for 35 min at 180 °C.

Figure 1b-c show scanning electron microscopy (SEM) and high-resolution transmission electron microscopy (HR-TEM) images of the as-developed nanogap, respectively. Additional images of the wafer-scale processing steps are presented in Supplementary Fig. 1**,** while Supplementary Fig. 2 displays the atomic force microscopy (AFM) images of the NGEs before and after ZnO deposition. The size of the self-aligned nanogap (L) formed between Au-Al varies between sub-20-nm to ~ 50 nm (Fig. 1c) with an average gap length of 24.7 nm as determined from SEM image analysis (Supplementary Fig. 3 and Supplementary Fig. 4). Crucially, analysis of the cross-sectional TEM image in Supplementary Fig. 5 reveals that ZnO fills the nanogap and provides good contact with M1 and M2 electrodes. Figure 1d shows a photograph of a fully processed 4-inch glass wafer containing a large number of nanogap Schottky diodes of varying geometries and topologies while the inset highlights a discrete interdigitated NGE-based ZnO diode.

**Fig. 2:**
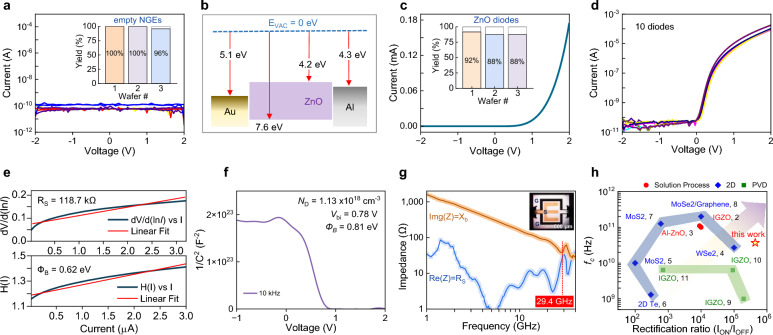
Electrical characterisation of the nanogap Schottky diode. **a**, I-V characteristics measured from 10 empty NGEs. The inset shows the manufacturing yield for the empty NGEs processed on three different wafers. **b**, Energy-band diagram of the NGE Au/ZnO/Al diodes before contact (i.e. no band bending). **c**, Representative linear I-V curve measured for a ZnO Schottky diode. The inset shows the yield of the ZnO nanogap Schottky diodes from three different wafers. **d**, semi-logarithmic plots of the I-V curves measured from 10 Schottky diodes. **e**, Cheung-plot used to calculate the barrier height and series resistance of the ZnO diodes. **f**, Representative Mott-Schottky plot from where the barrier height, built-in potential, and doping concentration were calculated. **g**, Real and imaginary impedance (Z) components versus frequency calculated using two-ports S-parameter measurements. The inset image shows the two microwave 500-µm-pitch Ground-Signal-Ground (GSG) probes and the ZnO diode under test. **h**, Intrinsic cut-off frequency versus current I_on/off_ values published in the literature and this work.

The manufacturing yield of the nanogap electrodes (NGEs) was assessed via I-V measurements performed prior to ZnO deposition. An important trait of the NGEs is the complete physical separation between the two metal electrodes and the absence of electrical shorts. Figure 2a shows the I-V traces from ten randomly selected empty NGEs fabricated on the same wafer. The current levels remain low (< 10^–10^ A) for all devices, indicating fully isolated electrodes. The inset in Fig. 2a shows the manufacturing yield obtained from devices fabricated on three different wafers, each having 24 NGEs. Yields of 100% (wafer-1), 100% (wafer-2) and 96% (wafer-3) were obtained highlighting the robustness of the manufacturing process even when performed under standard laboratory conditions. Depositing the ZnO atop the NGEs forms Schottky and Ohmic contacts with the Au and Al electrodes, respectively, due to their energetics (Fig. 2b)^33^. Figure 2c, d show the quasi-static I-V characterisation of ZnO nanogap diodes in linear and semi-log plots. The diodes exhibit a high on–off current ratio (4.4 × 10^5^ at 1 V) and a low reverse current of less than 10^–10^ A. To quantify the percentage of the functional diodes, we establish a yield criterion by considering as functional diodes only those exhibiting I_on/off_ of > 10^4^ at 2 V:

1 $$Yield = \frac{{\# diodes\,with\,I_{ON/OFF} \ge 10^{4} }}{\# total\,diodes\,measured} \times 100\%$$

The inset in Fig. 2c shows the calculated yields (92, 88 and 88%) for diodes manufactured on three different 4-inch wafers, while Fig. 2d presents the I-V curves for ten randomly selected devices, highlighting the similarity in their operating characteristics. Additional electrical analysis is presented in Supplementary Fig. 6. Specifically, Supplementary Fig. 6a shows the I_on/off_ as a function of absolute voltage for the diodes analyzed in Fig. 2d, in which a value of > 10^4^ was chosen as the rectifying condition threshold for the diodes. The extracted turn-on voltage of the diodes is ~ 0.55 V (I_on/off_ > 10^4^), following the above-mentioned criteria. The forward linear and semi-log plots of the I-V curves shown in Supplementary Fig. 6b reveal the different charge transport regimes, namely quantum mechanical tunneling, thermionic emission, and trap-assisted space-charge-limited current (SCLC) observed (Supplementary Fig. 6c)^34,35^. The thermionic emission process for a Schottky diode corresponds to a linear region in the semi-logarithmic I-V curve (Fig. 2d and Supplementary Fig. 6c) expressed as^36,37^:

2 $$I= {I}_{0}\text{exp}(\frac{eV}{nkT})\left[1-\text{exp}(-\frac{eV}{kT})\right]$$

3 $${I}_{0}=S{A}^{*}{T}^{2}\text{exp}(\frac{-e{\Phi }_{B}}{kT})$$

where *I*_*0*_ is the reverse bias saturation current, *n* is the ideality factor, *k* is the Boltzmann constant, *S* is the diode’s active area, A^*^ is the effective Richardson constant, and Φ_*B*_ is the barrier height for electron injection.

The quasi-static DC performance of the NGE ZnO diodes was further studied by analysing their responsivity and nonlinearity, two important parameters for practical applications (Supplementary Fig. 6d-e)^38^. For RF applications, the responsivity quantifies the diode’s ability to rectify an alternating current (AC) input signal to direct current (DC)^28,39^. As shown in Supplementary Fig. 6 d, the planar ZnO diodes exhibit a responsivity of 15 AW^−1^ at 0.12 V. The diode’s nonlinear response, on the other hand, indicates the deviation from the linear resistor-like behaviour and is defined as the ratio of the differential conductance (*dI/dV*) to the conductance (*I/V*) of the device. A nonlinearity value of over 3 is the typical boundary at which the diode reaches the ON state. Based on these criteria, the ON state for our NGE-based ZnO diodes is reached at around 0.2 V (Supplementary Fig. 6e). The ideality factor, *n*, on the other hand, accounts for the non-ideality related to single-carrier transport processes and, in practice, often has a value of > 1. Factors such as series resistance (*R*_*S*_), tunnelling currents through the barrier, interface states, and presence of insulating interfacial layers contribute to *n* >1^40^. The ideality factor of our NGE ZnO diodes was calculated from the slope of the linear region to be 1.75, while the barrier height for electrons (*Φ*_B_) was estimated at around 0.68 eV.

The *R*_*S*_is also a critical parameter and was calculated for the NGE ZnO diodes using the Cheung plot method^41^:

4 $$\frac{dV}{d(\text{ln}I)}= \frac{\eta kT}{q}+I{R}_{S}$$

Figure 2e depicts the plot of $\frac{dV}{d(\text{ln}I)}$ versus I, where an *R*_*S*_ = 118.7 kΩ is extracted from the slope of the linear fit. Additional details about the Cheung method are given in Supplementary Text 4b. Eq. 3 and 4 can be further modified to:

5 $$H(I) = V - (\frac{\eta kT}{q})\ln (\frac{I}{{SA^{*} T^{2} }}) = \eta \phi_{B} + IR_{S}$$

Eq. 5 provides an alternative method to calculate *Φ*_B_ from the intercept of the H(I) with the I axis, as shown in Fig. 2e (and Supplementary Fig. 7), yielding 0.62 eV. The latter value is in good agreement with the value of *Φ*_B_ = 0.68 eV obtained from the thermionic emission analysis. Capacitance–voltage (C-V) measurements at different frequencies provided further insights into the junction properties (Supplementary Fig. 8). Figure 2f displays the Mott-Schottky plot extracted from C-V measurements at 10 kHz. Using the data in the depletion bias regime, the junction’s built-in potential (V_bi_) and the dopant concentration (N_D_)^42^ were calculated, yielding 0.78 V and 1.13 × 10^18^ cm^−3^, respectively (Supplementary Table 1). The Mott-Schottky analysis shows that despite the planar architecture the NGE diodes exhibit qualitative similar operating regimes to traditional Schottky diodes.

To evaluate the dynamic response of the NGE ZnO diodes, their intrinsic cut-off frequency (${f}_{c}$) was evaluated from the input reflection coefficient (S-parameters) measurements using a two-port configuration (Supplementary Fig. 9). Figure 2g shows the imaginary [Img(Z) = X_b_] and real [Re(Z) = R_S_] impedance as a function of input signal frequency. The inset in Fig. 2g shows a photograph of the two-port NGE diode used for the S-parameter analysis developed using Coplanar Waveguide (CPW) design rules. In brief, to maintain a 50 Ω output impedance for a 300 µm wide signal electrode, 50 µm was calculated as the separation distance between the ground (G) and source (S) terminals, while 500-µm-pitch ground-source-ground RF probes were used to carry out the measurements following calibration in the range 0.3–40 GHz. The *f*_C_ was estimated as the frequency at which the X_b_ and R_S_ intersect, yielding a value of ≈29.4 GHz. This extraordinary cut-off frequency is attributed primarily to the planar structure of the NGE and the low capacitances^26,27,43^. We also found that the device size plays an important role, and a trade-off must be made depending on the targeted application. For example, NGE diodes with smaller channel widths (*W*) offer higher *f*_C_ but exhibit reduced current driving capabilities and responsivity^26^. To overcome these issues, diodes with interdigitated electrode layouts were developed to achieve higher forward current while maintaining a high *f*_C_. Figure 2h compares the I_on/off_ (1 V) and *f*_C_ of the nanogap diodes with those of state-of-the-art diodes reported in the literature (Supplementary Table 2). Evidently, our NGE ZnO diodes combine a remarkable *f*_C_ with high I_on/off_, making them an ideal building block for RF circuits.

**Fig. 3:**
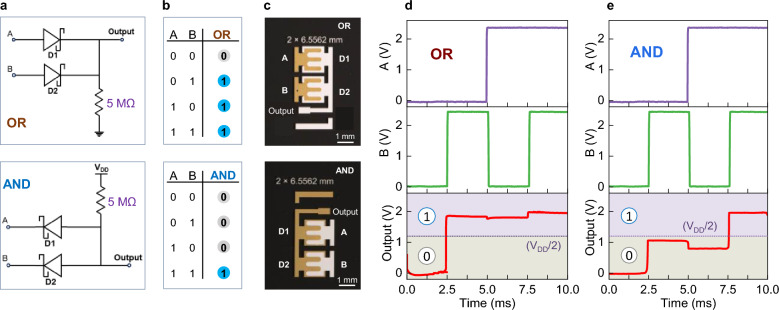
Diode-based logic gates. **a**, Schematics of the OR and AND logic gates circuitry. **b**, Truth table for OR and AND logic gates. A and B are the input signal states, and OR/AND is the Output. **c**, Photographs of the OR and AND logic gates developed. The NGE’s width for each NGE diode was 6.5562 mm. **d-e**, The real-time voltage waveforms of the inputs A & B and output signals for the OR and AND gates, respectively.

To assess the potential of NGE diodes for use in logic circuitry, we developed monolithic OR and AND gates. Figure 3a-b show the gate circuitry adopted and their truth tables, respectively. Figure 3c shows photographs of the NGEs diode based OR and AND gates manufactured onto 4-inch wafers. The definition of a logic state depends on the limits defined by the designer. A conservative convention to set a boundary between binary “1” and “0” states is to use (V_DD_/2) as a boundary threshold, where V_DD_ is the power supply voltage. An output value (Output) higher than V_DD_/2 defines the logic state of “1”, while an Output < V_DD_/2 defines the “0”.

For the OR gate (Fig. 3a), when both A and B inputs are set to “0”, there is no current passing through the diodes, and the output node is close to 0 V (ground level), representing a digital output of “0”. An output binary state of “1” is achieved whenever any or both of the inputs are set at logic “1”. Under these biasing conditions, the diode(s) is/are forward-biased with a small voltage drop across, compared to that across the load resistor, leading to a high output voltage. By adding more diodes in parallel, the number of logic inputs can be increased to accommodate multi-bit operations.

For the AND gate (Fig. 3a, bottom), a high output voltage (binary “1”) is only possible when both inputs are set to “1” (i.e. V_DD_). When a binary input signal of “0” is applied to one or both diodes, the low voltage drop across them results in a high voltage drop across the load resistor, leading to an output of “0”. To verify the functionality of these NGE-based ZnO gates, two square wave signals with an amplitude of 2.4 V (V_DD_) were used to define the binary input signals, while the output voltage was monitored in real-time using a digital oscilloscope. Both gates execute the intended logic operations successfully in accordance to their truth tables (Fig. 3b). The dynamic input/output waveforms for the AND and OR gates are presented in Fig. 3d-3e, respectively. Supplementary Fig. 10 displays additional results obtained at different input signal voltages, demonstrating operating stability at different biasing conditions. The transient response of the AND and OR logic gates at 2.4 V is displayed in Supplementary Fig. 11, for which the rise and decay times of the OR logic gate are around 12 µs and 300 µs, respectively; the rise and decay times for the AND logic gate are around 160 µs and 75 µs, respectively.

**Fig. 4:**
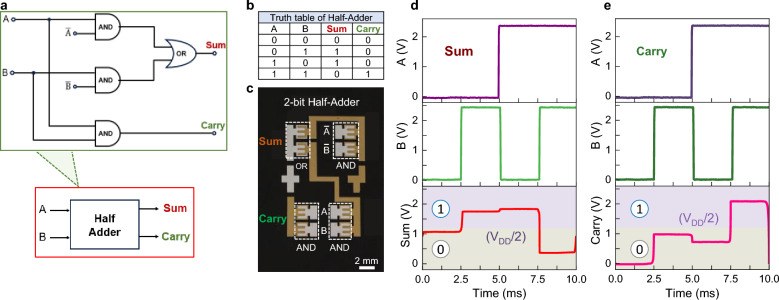
Monolithically integrated diode-based 2-bit half-adder. **a**, Circuit diagram and schematic symbol of the Half-Adder. **b**, The truth table of a 2-bit half-adder. **c**, Photograph of the fabricated 2-bit half-adder with all the I/O labelled. **d**, Voltage waveforms of the A and B inputs and the measured “sum” output of the Half-Adder. **e**, Representative voltage waveform of the A and B inputs and the measured “carry” output signals of the Half-Adder.

The feasibility of integrating NGE diode-based logic gates into more complex circuits was further demonstrated by developing a Half-Adder due to its fundamental role in ALUs. The circuitry and symbolic representation of the 2-bit Half-Adder and the corresponding truth table are shown in Fig. 4a-b. The circuit consists of three AND and one OR gates configured in two stages with two binary outputs, “Sum” and “Carry”. In the Full-Adder configuration, the carry output of the circuit (Fig. 4a) is transferred as the third input to the next bit order Half-Adder. Supplementary Fig. 12 and Supplementary Text 6 provide information on decimal addition and operation for more detailed description. Figure 4c displays a micrograph of the monolithic NGE ZnO diode-based 2-bit Half-Adder circuit developed. The operating conditions used were the same as those for the OR and AND gates in Fig. 3. The measured sum and carry outputs are presented in Fig. 4d-e, demonstrating Half-Adder functionality in accordance with its truth table (Fig. 4b). The circuit remains functional under different biasing conditions (Supplementary Fig. 11), demonstrating robust operation.

In conclusion, we developed planar nanogap ZnO diodes for arithmetic logic applications. The manufacturing process developed is scalable and delivers functional diodes with a yield of ≥ 98%. Discrete diodes exhibited low turn-on voltage, high on–off current ratios (> 10^6^ at 2 V), low reverse currents (< 100 pA), and an impressive cut-off frequency of over 25 GHz. Crucially, the diode’s unique planar geometry enables monolithic integration into diode-based logic circuits, such as AND and OR gates. By integrating several of these logic blocks, we demonstrated a 2-bit Half-Adder, a fundamental component of modern arithmetic logic units. The Half-Adder shows robust two-bit logic operation under different bias conditions. Compared to existing circuit development approaches, our diode-based technology provides a simpler and scalable manufacturing path for large-area monolithic RF circuits for application in the emerging 5G and 6G sectors.

## Methods

### Fabrication of ZnO nanogap Schottky diodes

Borofloat 4″ 1.1 mm thick glass wafers (from Semiconductor Wafer Inc.) were sequentially cleaned with acetone, IPA and de-ionized (DI) water baths under sonication for 5 min each. A 1.7 µm thick layer of AZ 5214E photoresist (PR) was spin-coated on the glass wafer and patterned via photolithography and developed with AZ 726 MIF (from MicroChemicals). A 100-nm-thick layer of Al was deposited by thermal evaporation on the patterned PR (by brightfield mask) at $1\times {10}^{-6}$ mbar at a rate of 0.5 Å/s and developed by overnight liftoff in acetone to pattern the metal layer 1 (M1). A solution containing 1.5 mM of ODPA (Sigma Aldrich) in 80 ml of IPA was prepared for the self-assembled monolayer (SAM) deposition. Substrates were dipped in the solution for 24 h to form a SAM on the patterned Al electrodes. A couple of cycles of IPA rinsing and drying in a nitrogen gas stream were carefully applied to the sample to remove any excess ODPA; this was followed by annealing the samples at 80 °C for 15 min in air to remove residual solvent. Next, a 5-nm Al layer was deposited by thermal evaporation, followed by a 95 nm Au layer on top to form the second metal layer (M2) at a rate of 0.5 Å/s each; the thin bottom Al layer promotes the adhesion of the Au layer to the substrate. By dipping the substrate in N-Methyl-2-pyrrolidone at 88 °C, the M2 areas overlapping SAM on M1 are selectively removed as a consequence of reduced adhesion of M2 on the M1/SAM surface. This removal process creates the nanogap between M1 and M2. Afterwards, a second photolithography process is carried out to realize the final shape of the Al electrodes and contact pads (see Supplementary Fig. 1), using a Brightfield Mask with AZ 5214E after the exposure and MIF726 development. Two sets of two photomasks each (M1 patterning by lift-off, M1 patterning by wet-etching) were used in total (2 masks each): One set for the sample fabrication of the 2-Ports nanogap Schottky diodes and OR and AND gates, and another set of photomasks for the fabrication of the half-adder samples. The substrate is immersed in Al etchant (from Alfa Aesar) for 4 min, then washed in water, followed by a sequential sonication cleaning using acetone, IPA and DI water (5 min each). The remaining SAM on the Al surface is removed by exposing the sample to an argon plasma for 10 min, which provides the empty nanogap structure. To deposite the ZnO semiconductor layer, 120 mg of (ZnO, from Sigma Aldrich) were initially dissolved in 12 ml of ammonium hydroxide (50% v/v aq. soln. from Alfa Aesar) during at least 18 h of stirring. Next, a first layer of ZnO was deposited on the sample spin-coating at 3000 RPM for 40 s and annealing at 180 °C for 5 min. Next, a second ZnO layer was obtained by spin-coating at 4000 RPM for 60 s and annealing at 180 °C for 30 min. Finally, 5 MΩ 0603 SMD-Resistors were manually connected to the logic gate (OR and AND gates) output by applying conductive silver paste by Electron Microscopy Sciences (EMS) between the resistor electrodes and the contact paths, and annealing them for 15 min at 60 °C. 2 and 20 MΩ resistors were similarly added to the half-adder gates, using the same annealing steps: A 2 MΩ 0603 SMD-Resistor was attached by silver paste to each one of the two AND gates of the first Sum Stage, while a 20 MΩ 0603 SMD-Resistor was attached to the OR and AND gates of the Sum and Carry stages, respectively.

### Imaging characterisation

Atomic Force Microscope (AFM) images of the Al/Au empty nanogap structures were obtained with a Bruker Dimension Icon AFM. Additionally, scanning electron microscope (SEM) top-view images were obtained by using a Helios 5 UX (Thermo Fischer Scientific) with a field emission electron source working at 5 kV. For the cross-sectional images of the Al/Au Nanogap structure, the focused ion beam (FIB) mode of the Helios UX SEM was used: A 300 nm Carbon layer was deposited to protect the Nanogap structure, followed by a 1-µm-Tungsten-layer deposition. The samples were then tilted, and a Gallium ion beam was used to etch around 6–8 µm at the sampling section edge. The High-Resolution TEM (HR-TEM) cross-sectional view was obtained by using a field emission electron source at 30 kV at a beam current of 41 pA on the sample tilted 52°.

### Electrical and frequency characterisation

Current–Voltage (IV) measurements were performed using a Keysight B2912A source/measure system in a nitrogen-filled glovebox. Capacitance measurements were undertaken using a Keysight B1500A Semiconductor Device Analyzer at 10 kHz, 100 kHz and 1 MHz while the samples were maintained at room temperature inside a nitrogen-filled glove box. High-frequency scattering parameter measurements for two-port devices (${S}_{11}, {S}_{12}, {S}_{21}, {S}_{22}$) were carried out in air using a Cascade Infinity GSG station through GSG probes ACP-40 with 500-µm pitch. The probes were connected to an Agilent N5225A Vector Network Analyzer, measuring a frequency range from 300 MHz to 40 GHz. The input signals were generated with a signal generator Tektronix AFG 3252 Dual Channel Arbitrary/Function Generator, generating signals from 50 to 500 Hz, as well as input voltage peak values in the range 1.2–4 V. The transient response of the AND and OR gates, as well as that of the half-adder, was measured with a Tektronix Mixed Domain Oscilloscope MDO4104C.

## Supplementary Information

Supplementary Information.

## Data Availability

The datasets used and/or analysed during the current study available from the corresponding author on reasonable request.
