# Supplementary material for: Wafer-scale radio frequency ZnO Schottky diodes and arithmetic circuits
Source: Sci Rep. 2025 Jul 8;15:24338. doi: 10.1038/s41598-025-06506-8 (PMC12234897; doi:10.1038/s41598-025-06506-8)
Supplement: Supplementary file 1 — Supplementary Information. [file 41598_2025_6506_MOESM1_ESM.docx]

**Supplementary Information**

**Wafer-Scale Radio Frequency ZnO Schottky Diodes for Arithmetic Circuits**

Harold F. Mazo-Mantilla*(1), Zhanibek Bizak* (2), Linqu Luo (1), Hendrik Faber (1), Camelia Florica (3), Suman Mandal (1), Atif Shamim (2), Khaled N. Salama (2) †, and Thomas D. Anthopoulos (1)(4) †

(1) King Abdullah University of Science and Technology (KAUST), KAUST Solar Center, Materials Science and Engineering Department, Physical Science and Engineering (PSE) Division, Thuwal, Makkah, 23955, Saudi Arabia

(2) King Abdullah University of Science and Technology (KAUST), Electrical and Computer Engineering Department, Computer, Electrical and Mathematical Sciences and Engineering (CEMSE) Division, Thuwal, Makkah, 23955, Saudi Arabia

(3) King Abdullah University of Science and Technology (KAUST), Nanofabrication Core Lab (NCL), KAUST Core Labs, Thuwal, Makkah, 23955, Saudi Arabia

(4) Henry Royce Institute, Photon Science Institute, Department of Electrical and Electronic Engineering, The University of Manchester, Manchester M13 9PL, UK.

*These authors contributed equally.

†Corresponding authors: [thomas.anthopoulos@manchester.ac.uk](mailto:thomas.anthopoulos@manchester.ac.uk); khaled.salama@kaust.edu.sa

**This word file includes:**

Supplementary Texts **1-6**

Supplementary Figures **1-11**

Supplementary Tables **1-2**

Supplementary References **1-15**

**Supplementary Texts**

**ST 1. Self-assembled monolayer (SAM) formation for Al (M1) and Al/Au (M2) electrodes**

The nanogap formation between two metal electrodes is reached by the development of a Self-Assembled Monolayer (SAM) between them. In this work, Aluminum (Al) was patterned as first metal layer (M1), while a bi-layer of Aluminum-Gold (Al/Au) was deposited as second metal layer (M2), in which the Al works as an adhesion layer to the Au layer. The SAM molecule used in this research was Octadecylphosphonic Acid (ODPA), which is characterized for being able to be attached on native oxide metal surfaces such as Al_2_O_3_ (on Al layers) or TiO_2_ (on Titanium, Ti, layers). The Nanogap formation process, including the step of ODPA SAM deposition on patterned M1, is shown in **Supplementary Fig. 1** by schematics and pictures, to illustrate how the sample is developed during the process. The process is described starting with the physically adsorbing attachment of the ODPA head groups (-POOH) to the native oxide on the M1 native oxide surface (AlO_x_); this SAM deposition is carried out by a chemical bonding on M1 surface, while being facilitated by the solvent evaporation (Isopropanol, IPA, in this work). The ODPA chain tail (-CH_3_) creates a hydrophobic surface on the SAM layer, which reduces the adhesion capability of M2 on the M1/SAM layer surface. This lower adhesion promotes the nanogap development by preventing the M2 bonding with M1 in the areas where they overlap each other.

**ST 2. Geometric description of nanogap electrodes**

The nanogap structure developed for this work is based on co-planar interdigitated electrodes (IDE), as shown in the final fabrication process picture in **Supplementary Fig. 1**. For these devices, the **Supplementary Scheme 1** depicts a geometrical scheme of the nanogap electrode structure, in which *L* is the nanogap channel length and it is defined as the distance between the electrodes (≤ 30 nm for this work). *H* is defined as the electrode height, it is, the thickness of layers M1 and M2 (100 nm for this work). The nanogap channel width, *W*, is defined as the nanogap channel perimeter (length of red path in **Supplementary Scheme 1**), measured as the distance along which both electrodes face each other (6.5562 mm for the Schottky Diodes used in this work).

**Supplementary Scheme 1 |** Illustration of the Nanogap dimensions.

**ST 3. Average nanogap channel length extraction**

The characterization of the nanogap fabrication includes the measurement of the mean channel length L and its distribution (**Supplementary Fig. 3**). This was carried out by analyzing 3 SEM images (see **Supplementary Fig. 4,** which depicts the three analyzed samples). The gap length L was extracted manually in multiple positions across the image with a horizontal separation of 4-6 nm between adjacent measurement positions (see vertical blue bars in **Supplementary Fig. 4g-i**).

**ST 4. Figures of merit for nanogap Schottky Diodes**

There is a plethora of key performance characterization parameters for RF Schottky devices and relative metrics of methods that are used to check viability of method/device, to assess the utility for potential applications or to do comparative analysis relative to alternatives. Below, it is shown a discussion about important figures of merit and performance metrics of Schottky diodes for use in diode logic and for 5G applications.

**ST 4.a. On-off current ratio, non-linearity, and responsivity of the Schottky diodes**

The on-off current ratio (I_on/off_) is defined as the forward-current to reverse-current ratio as a function of the (absolute) voltage; this allows to quantify the diode asymmetry^[1-2]^. The I_on/off_ as a function of (absolute) voltage for the 10 nanogap Schottky diodes in manuscript **Fig. 2d** is shown in the **Supplementary Fig. 6a**; this shows that the nanogap Schottky diodes reach an on state (I_on/off_ >${10}^{4}$) around 0.55 V.

$I_{on/off} = \left| \frac{I_{F}(V)}{I_{R}(-V)} \right|$ (S1)

The Schottky diode non-linearity describes how deviated the device is from the behaviour of an ideal linear resistor. The non-linearity as a function of the voltage is described as the ratio of the differential conductance (*dI/dV*) to the conductance (*I/V*)^[1-2]^ (**Supplementary Fig. 6e**):

$\mathrm{Nonlinearity}=\frac{\frac{dI}{dV}(V)}{\frac{I(V)}{V}}$ (S2)

The Quasi-DC responsivity (**Supplementary Fig. 6d**) quantifies the change of the device DC output current as a function of the RF input power. The small-signal approximation from the I-V behaviour is used to calculate the device responsivity (ratio of the second derivative of the I-V to the differential conductance^[1, 3]^):

$\mathrm{Responsivity}=\frac{1}{2}\frac{\frac{d^{2}I}{dV^{2}}(V)}{\frac{dI}{dV}(V)}$ (S3)

**ST 4.b. Resistance, ideality factor and free carrier density calculation**

The diode series resistance (R) is obtained from the I-V following the Cheung method, in which the relevant voltage range was determined from the log I-V plot as the area enclosed by the intersect of the two linear regions (**Supplementary Fig. 7b**). Starting with Cheung formulas:

$V=R_{S}I+\eta\Phi_{B}+\frac{\eta kT}{q}ln(\frac{I}{AA^{*}T^{2}})$ (S4)

$\frac{dV}{d(\ln I)}= \frac{\eta kT}{q}+IR_{S}$ (S5)

In which $R_{S}$ is the series resistance, $\Phi_{B}$ is the barrier height, $\eta$ is the ideality factor, $k$ is the Boltzmann constant, $T$ is the temperature in Kelvin (K), $q$ is the electron charge in (C), A is the diode area (cm^2^) and A^*^ is the effective Richardson constant (32 A/cm^2^ K^2^ for ZnO) . From the S5, the series resistance is obtained as the function slope. Consequently, we plot the next function:

$H\left( I \right)=V-$($\frac{\eta kT}{q})ln(\frac{I}{SA^{*}T^{2}})$ = η$\Phi_{B}+IR_{S}$ (S6)

By plotting H(I)=f(I) and by a linear fitting, it is possible to obtain a second approximation of R_S_ from the slope, while $\Phi_{B}$ is obtained from the intersect. The Cheung plots based on the Eq. (S6) are shown in **Supplementary Fig. 7c**. For these calculations, the diode geometrical area was taken into account, after neglecting the structure edge effects. Since the Cheung equations provide a few accurate ideality factor ($\eta$), an additional more-accurate ideality factor was obtained from the log I-V plot, as shown in **Supplementary Fig. 7a** and in the **Supplementary Table 1**, based on the thermionic emission model (Shockley diode equation) for low voltage (space-charge limited) regime:

$I= I_{0}exp(\frac{\mathrm{eV}}{\eta kT})\left[ 1-exp(-\frac{\mathrm{eV}}{\mathrm{kT}}) \right]$ (S7)

$n= {(log}_{10}e)\times\frac{q}{slope\times kT}$ (S8)

**ST 4.c. Schottky barrier height and dopant concentrations**

C-V measurements provide alternative methods to obtain the Schottky barrier height. This approach is a practical way to determine the value of flat band barrier height, while providing negligible lowering image force effects ^[4]^. The Mott-Schottky plot, 1/𝐶^2^ = *f*(𝑉), is used to calculate the built-in voltage, V_bi_ (voltage for no band bending), the charge depletion separating the depletion and the accumulation regions, and the dopant concentration N_D_. The extrinsic capacitance due to 3D coupling of the electrodes without semiconductor material (empty inter-electrode gap) has been subtracted from raw data (0.17 pF) to assure that the measured capacitance values are obtained mostly from the final device, as described by the next equation:

$\frac{1}{C^{2}}=\frac{2(V_{bi}-V-\frac{kT}{q})}{A^{2}q\varepsilon\varepsilon_{0}N_{D}}$ (S8)

In which ε is the dielectric constant of ZnO, and ε_0_ is the dielectric permittivity in vacuum (ε_0_ = 8.856×10^-12^ F m^-1^). The Mott-Schottky plot (**Supplementary Fig. 8**) provides another method to determine $\Phi_{B}$ and its value can be derived as:

$\Phi_{B}=V_{bi}+\frac{kT}{q}(ln\frac{N_{CB}}{N_{D}}+1)$ (S9)

Where N_CB_ is the effective density of states in the conduction band, being calculated as:

$N_{CB}={2(\frac{2\pi m^{*}kT}{h^{2}})}^{3/2}$ (S10)

For ZnO, given m* = 0.27m_0_^16^, the N_CB_ and $\Phi_{B}$values are calculated as 3.5×10^18^ cm^-3^ and 0.810 eV respectively.

**ST 4.d. Cut-off frequency estimation**

The Schottky diode cut-off frequency ($f_{c}$) is can refer to an intrinsic or an extrinsic cut-off frequency value, depending on the measuring and extraction method of the $f_{c}$ ^[5]^. The intrinsic cut-off frequency ($f_{c,int}$), measured via 2-Ports S-Parameters (Scanning Parameters, **Supplementary Fig. 9**) reflection/transmission/isolation measurements, includes the losses associated to the device and represents a theoretical upper limit. $f_{c,int}$ can be theoretically extracted via examining the equivalent circuit model as shown in the **Supplementary Scheme 2**.


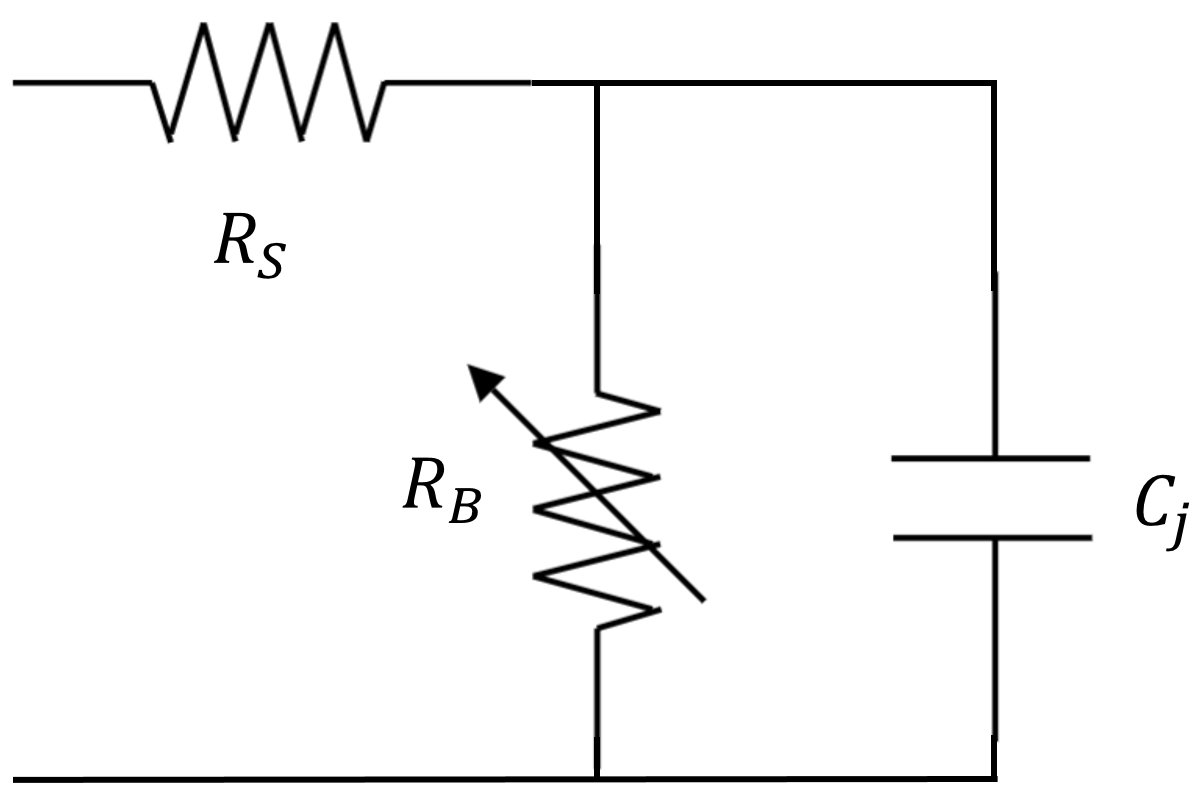


**Supplementary Scheme 2 |** Equivalent circuit model for a Schottky diode, showing series resistance (R_S_), parallel junction capacitance (C_j_) and barrier resistance (R_B_).

The equation given for the output voltage of this RC circuit is described as:

$V_{\mathrm{out}}=V_{\mathrm{in}}\frac{X_{C}}{\sqrt{R_{S}^{2}+X_{C}^{2}}}$ (S11)

In which the series resistance, R_S_, is set in series with nonlinear barrier resistance R_B_, and the X_C_ is the reactance associated with the junction capacitance (C_j_), described as:

$X_{C}=\frac{1}{wC_{j}}=\frac{1}{2\pi fC_{j}}$ (S12)

Where f is the frequency in the equation S12. From equation S12, low frequencies ($f$) can be obtained when X_C_ >> R_S_, considering that resistive elements mainly dominate the current transport and the signal rectifying. Additionally, higher frequencies can be obtained when Xc << Rs due to the shorted current flow through the capacitive element, in addition to the decreased rectification at high frequencies. The threshold frequency at which the decreased rectification happens is defined as the intrinsic cut-off frequency, defined by the condition X_C_ = R_S_. From$f_{c}$, the RC constant can be determined using:

$f_{C}=\frac{1}{2\pi R_{S}C_{S}}$ (S13)

According to Eq. S13, the intrinsic *f*_C_ of the Schottky diodes depends on R_S_ and Cj. The series resistance, R_S_, combines the intrinsic semiconductor resistance (R_SP_) and the Ohmic and Schottky contact resistances between metal and semiconductor RC (R_ohmic_ and R_Schottky_). R_S_ is calculated from either static I-V measurements using a method proposed by Cheung et al.^[6]^ or from two-ports S-parameters dynamic reflection and transmission tests. Nonetheless, the series resistance and capacitance values are highly dependent on frequency, which provides more reliability and makes them widely useful to extract the diode impedance as well as to measure its intrinsic cut-off frequency^[7-8]^. In the present work, the real and imaginary components of the nanogap Schottky diode impedance were extracted from the two-ports S-Parameters measurements (**Supplementary Scheme 3**) as shown in **Fig. 2g** caption, and were plotted as function of frequency.


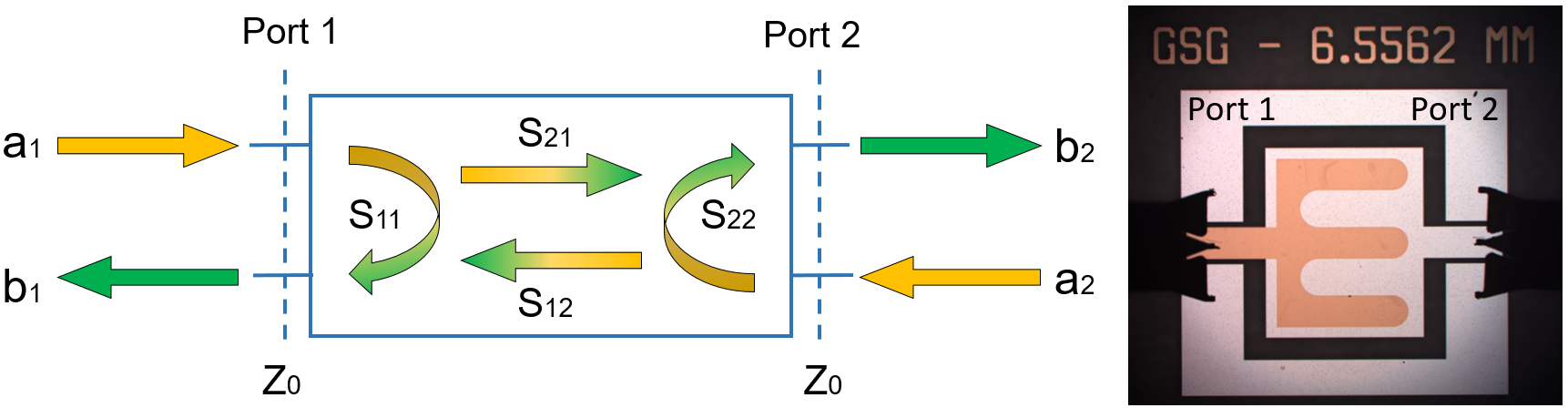


**Supplementary Scheme 3 |** S Parameter representation for a two-ports network, where: a_n_ is the input power at port “n”, b_n_ is the reflected signal at port “n”, Z_0_ is the reference impedance, S_11_ is the input reflection coefficient, S_12_ is the reverse voltage gain, S_21_ is the forward voltage gain and S_22_ is the output reflection coefficient. The right image shows the measuring RF probes on a nanogap Schottky diode sample to obtain its two-ports S-Parameters.

For the two ports network, the relation between the input and output parameters (a_n_ and b_n_) and the four S-parameters is defined as:

$$\left[ \begin{matrix} b_{1} \\ b_{2} \end{matrix} \right]=\left[ \begin{matrix} S_{11} & S_{12} \\ S_{21} & S_{22} \end{matrix} \right]\times\left[ \begin{matrix} a_{1} \\ a_{2} \end{matrix} \right]$$

Considering the parameters described by the two-ports network S-parameters, it is possible to convert the S-Parameter matrix to Z-parameter matrix as follows:

$$Z=\left[ \begin{matrix} Z_{11} & Z_{12} \\ Z_{21} & Z_{22} \end{matrix} \right]=\frac{Z_{0}}{D}\left[ \begin{matrix} \left( 1+S_{11} \right)\left( 1-S_{22} \right)+S_{12}S_{21} & 2S_{12} \\ 2S_{21} & \left( 1+S_{22} \right)\left( 1-S_{11} \right)+S_{12}S_{21} \end{matrix} \right]$$

$$D=\left( 1-S_{11} \right)\left( 1-S_{22} \right)-S_{12}S_{21}$$

The input impedance is $Z_{11}=Z_{in}$, for which $Z_{in}=R_{s}+jX_{c}$, as mentioned before ($Z_{0}=50 Ω$ was a design condition for the measurements, following the Coplanar Waveguide, CPW, design rules). This value is plotted as a function of frequency, and the cutoff frequency ($f_{c}$) is obtained where the real and imaginary impedance components coincide^[7, 9]^ ($R_{s}=|X_{c}|$). To find the application frequency condition, the device cut-off frequency must match the supplied input RF signal for at least 90% of the input RF signal. Nevertheless, the frequency response conditions for the device to match the supplied signal depends also strongly on the device loses by EM compatibility and circuit matching, which has high effects on the device rectifying performance.

**ST 5. Fundamental diode based AND and OR logic Gates operation**

Manuscript **Fig. 3a** depicts the schematic diagrams of OR and AND gates. OR gate electrical equilibrium equations for diodes D1 and D2 are given as

$V_{A}-V_{D1}= V_{Output}={(I}_{D1}+I_{D2})R$ (S14)

$V_{B}-V_{D2}= V_{Output}=(I_{D1}+I_{D2})R$ (S15)

The voltage and current are considered positive in voltage bias of the diode, namely when positive current is passing from anode to cathode. In case, when both input voltages are 0V (logic low feeding), the current through diodes D1 and D2 are zero, $I_{D1}=0A$ and $I_{D1}=0$A, and voltage output will be zero as well representing digital logic output “0”. When input A is V_DD_ and input B is 0V, D1 will be turned on while D2 will be turned off. Accordingly, $I_{D1}=\frac{V_{DD}-V_{D1}(I_{D1})}{R}$ and $I_{D2}=0A$ will be the electrical equilibrium condition and voltage at the output $V_{Output}=V_{DD}-V_{D1}(I_{D1})$. Since $V_{D1}(I_{D1})$ is on voltage of the Nanogap diode and considerably lower than $V_{DD}$, the voltage level at the output will be high, representing digital logic output “1”. The same analysis can be done when input A is 0V and input B is V_DD_ or when both inputs are biased with V_DD._ In these cases, the output will be $V_{Output}=V_{DD}-V_{D}(on)$ and will represent logic “1”. The truth table (**Fig. 3b**) summarizes the logic behavior of the OR gate.

Similar analysis can be done for AND gate, where electrical equilibrium equations for diodes D1 and D2 are given as

$V_{A}-V_{D1}= V_{Output}={(I}_{D1}+I_{D2})R$+$V_{DD}$ (S16)

$V_{B}-V_{D2}= V_{Output}=\left( I_{D1}+I_{D2} \right)R+V_{DD}$ (S17)

It is worthy to note that we are keeping the same vector notations for diodes and voltages, considering them positive when current flowing from anode to cathode. In first case, when both input voltages are low, the diodes D1 and D2 will be reverse biased having negative current passing from equal to saturation current in accordance with IV characteristic as in **Supplementary Fig. 6b**. Therefore, the voltage at the output will be $V_{Output}= V_{DD}- \left( I_{01}+I_{02} \right)R$ = $-V_{D1}= -V_{D2}$. When properly designed, the output voltage will scale toward 0V and represents digital logic output “0”. Similar analysis can be done when only one input is biased with V_DD_ while other input is biased with 0V. The output voltage will be $V_{Output}= V_{DD}- \left( I_{D1|D2} \right)R$ and will represent digital logic “0”. The final case, when both inputs are biased with V_DD_, the whole circuit will be in equilibrium with V_DD_ at every node and zero current. All logic states of the AND gate output for various input combinations are depicted in manuscript **Fig. 3b** as the truth table. The measurement results of the implemented diode logic based on nanogap Schottky diode are shown in **Supplementary Figure 10** for various bias voltages, namely 2.0 V, 2.4 V and 2.8 V. The performance of the diode logic gates improves with increasing bias voltages, as expected.

**ST 6. Diode based 2-bit Half-Adder operation**

The Arithmetic Logic Unit (ALU) is the computational brain of computers and digital circuits. ALU does complex computational tasks by executing series of basic arithmetic and logic operations, such as addition, subtraction, comparison, binary shifting. The key arithmetic circuit in any ALU is the Adder, which is used to do the binary addition of two binary inputs. The building block of Adder is the Half-Adder, which takes two single-bit inputs and computes their addition as an output. The “Sum” output of the Half-Adder is the least significant bit (LSB) of the output result, while the “Carry” output is the most significant bit (MSB) of the addition result, which indicates if there was a carry-over from the addition of the two inputs. The Half-Adder is constructed using 3 AND and 1 OR logic gates, and its schematic is shown in manuscript **Fig. 4a**. The Sum computation is done in two stages, where first stage AND gates take as an input A and $\bar{B}$ for the top AND gate, and B and $\bar{A}$ for the bottom AND gate. This combination is known as XOR and logic output is “1” when only when two inputs are complementary, meaning when inputs are combinations of logic high and low states. In other cases, when both inputs are logic high or logic low, the output will be logic “0” accordingly. The carry-over is computed with single AND gate, having “1” as Carry only when addition of the binary 1 and 1 as inputs. The truth table of the Sum and Carry output is depicted in **Supplementary Scheme 4** below. For readers convenience the binary truth table is also transformed to decimal addition. It is important to remark that binary number a_n_a_n-1_…a_3_a_2_a_1_ converted to decimal number as D = a_n_×2^n-1^ + a_n-1_×2^n-2^+…+a_3_×2^2^+a_2_×2^1^+a_1_×2^0^. Therefore, decimal 0 is 00 in binary, decimal 1 is 01 in binary and decimal 2 is 10 in binary_._


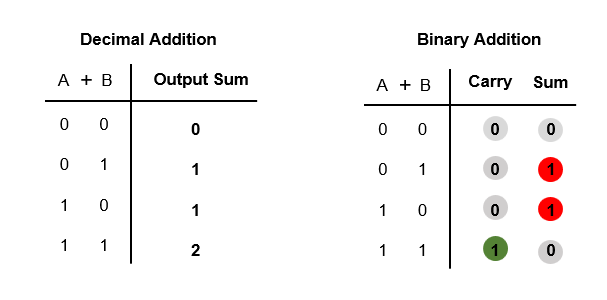


**Supplementary Scheme 4 |** The Half-Adder addition summarized in decimal and binary numbers.

The **Supplementary Figure 11** shows the measurement results of the implemented 2-bit Half-Adder for different bias voltages, namely for 2.0 and 2.4 V. Both measurements results agree with the truth tables shown in **Supplementary Scheme 4**.

**Supplementary Figures**

**Supplementary Figure 1 | Fabrication steps of Nanogap co-planar electrodes.** Schematic depiction of the fabrication steps to obtain several Nanogap co-planar electrodes on a large-substrate and corresponding pictures of actual wafers: (1) Thermal evaporation and patterning by photolithography and in-acetone lift-off of Al electrodes with the designed geometries, dipping of the Al-patterned substrate in octadecylphosponic acid (ODPA) solution in Isopropanol (IPA) and ODPA attachment stimulated by annealing on the Al native oxide (AlOx), (2) thermal evaporator deposition of Au electrodes on the Al/ODPA and empty-substrate areas, (3) Nanogap development by chemical removal of the Au layer areas overlapping the Al/ODPA patterns by dipping the sample in NMP and surface cleaning by exposing the substrate to Argon plasma, (4) empty Al/Au-Nanogap Schottky Diode structure after wet-etching to pattern the Al excess on the substrate.

**Supplementary Figure 2 | Atomic Force Microscope (AFM) image of the nanogap.** The picture depicts the nanogap structure from AFM height images. (a) Image of an empty Al-Al/Au nanogap structure, where there is a clear visual difference between the Al and Au shallow deposited grains after the whole fabrication process; (b) Image of an Al-Al/Au nanogap covered with ZnO, in which it is seen that the ZnO layer covers the Al and Au grains.


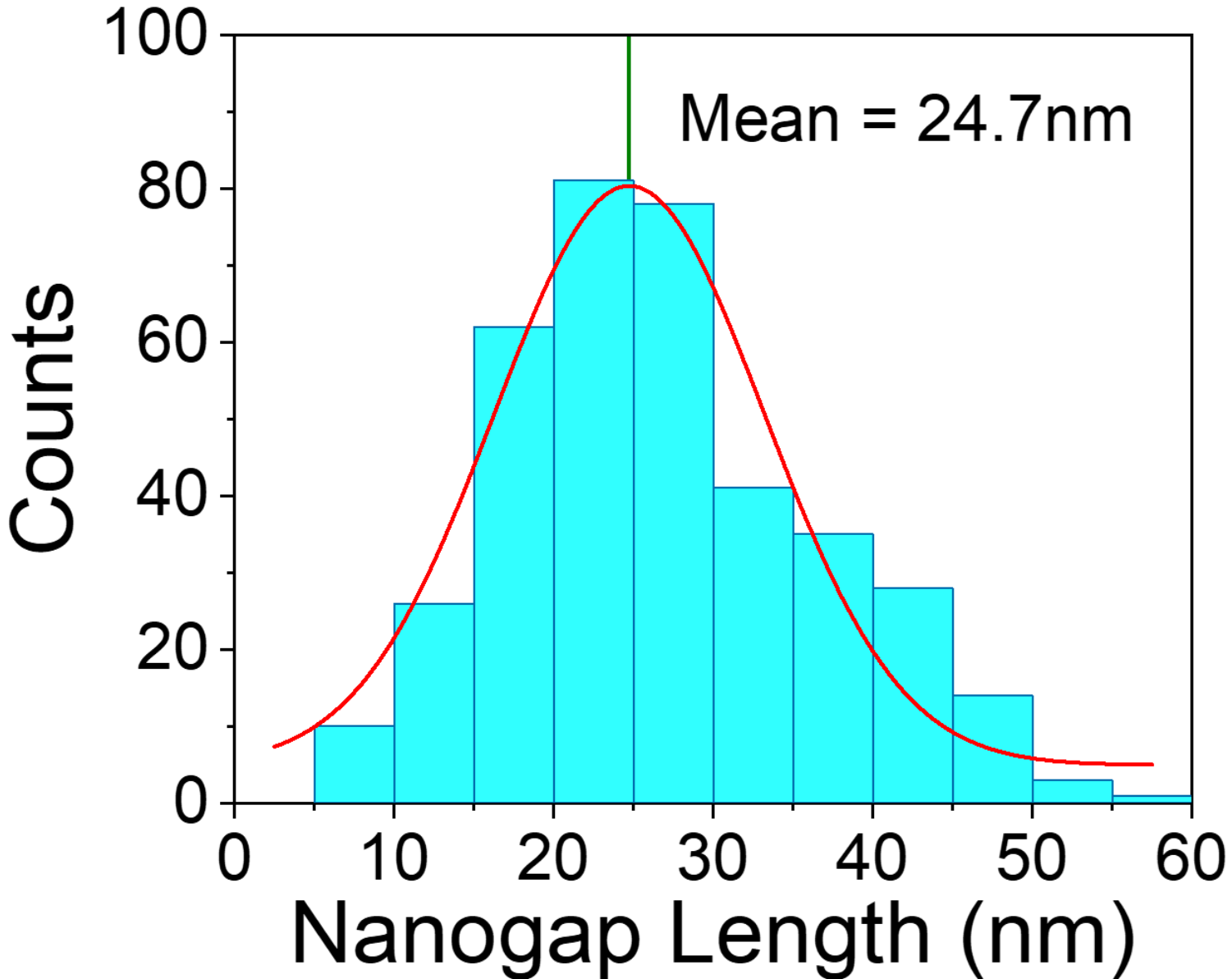


**Supplementary Figure 3 | Nanogap structure length statistics.** Statistical distribution for the nanogap sampling length values taken along three SEM images. The mean value out of the sampled values is 24.7 nm.

**Supplementary Figure 4** | Data sampling for the length extraction of a Nanogap structure sample. a), b) and c) SEM images used to extract the nanogap mean length, as described in Supplementary Text 3. d), e) and f) Overlay of the SEM image and locations where the gap length was measured. g), h) and i) Isolated display of the measured lengths along the gap.

**Supplementary Figure 5 | Cross Sectional analysis of the nanogap Schottky diode.** By using a FIB technique in the SEM, a portion of nanogap ZnO Schottky diode was checked from its cross-section by a HR-TEM. (a) Obtained TEM image of the nanogap cross section on a Si substrate, to be used for EDX material composition analysis; (b) EDX material distribution of the sample composition shown in image (a), showing the location of the electrode metals used in the test with a Ga beam (Al and Au).

**Supplementary Figure 6 | Quasi-static IV characteristics and electron transport regimes**. (a) I_on/off_ as a function of voltage (absolute) for 10 nanogap Schottky diodes, in which I_on/off_$={10}^{4}$ sets the minimum boundary for reaching a rectifying condition. (b) The IV characteristics of the nanogap Schottky diode in semi-log and linear scale. The force probe was on gold electrode whole ground probe on aluminum electrode during IV measurements. (c) Forward IV characteristic in log-log scale with highlighted electron transport regimes. Namely, region I, where slope is < 1, indicates tunneling as the dominant charge transport mechanism. Region II, in which exponential slope with increase bias voltage, indicates the prevalence of the thermionic emission mechanism. In region III, a very large slope indicates trap-assisted space-charge-limited current as the predominant charge transport mechanism. (d) The quasi-DC current responsivity plot. (e) The non-linearity curve as a function of voltage. The non-linearity is a measure of the deviation from a linear resistor and is defined as the ratio of the differential conductance (*dI/dV*) to the conductance *(I/V*). A non-linearity > 3 (marked by the green area in the graph) is preferred for high performing RF diodes.

**Supplementary Figure 7 | Schottky diode parameter extraction from IV characteristics**. (a) The ideality factor is extracted from the linear fit slope in the thermionic emission region from the semi-log IV plot (b) The region used for Cheung-plots in the forward-bias region (c) The dV/dlnI vs current (top graph) and the H(I) vs current plots for series resistance and the barrier height calculation form the Cheung-plot region.

**Supplementary Figure 8 | Capacitance – Voltage (CV) measurements and parameter extraction.** (a) The nanogap Schottky diode CV measurements from -2 V to 2 V at various frequencies. (b) The Mott-Schottky plot for nanogap Schottky diode at 100 kHz, where red curve represents the raw measurement (before correction) and the blue curve represents the data after a correction by subtraction of the corresponding extrinsic empty gap capacitance where no semiconductor material was present. (c) The Mott-Schottky plot for all three frequencies after the correction of extrinsic capacitance was performed. (d) The extraction of the built-in voltage (V_bi_) from the intersection of the linear fit with x-axis, barrier height ($\Phi_{B})$ and of the donor concentration (N_D_) from the slope of the linear fit.

**Supplementary Figure 9 | 2-Ports S-Parameters results for a Nanogap Schottky diode device.** S-parameter results after a 2-ports testing on a Nanogap Schottky diode: (a) S_11_; (b) S_12_; (c) S_21_; (d) S_22_.

**Supplementary Figure 10 | OR and AND diode logic gate measurements.** (a) Diode-based OR gate measurements at various input signal voltages and V_DD_ (2, 2.4, 2.8 V). (b) Diode-based AND gate measurements at various input potentials and V_DD_ (2, 2.4, 2.8 V).

**Supplementary Figure 11 | Response times of AND and OR logic gates.** Rise and decay response times of OR (a) and AND (b) logic gates at 2.4V.

**Supplementary Figure 12 | 2-bit Half-Adder arithmetic circuit measurements.** Input signal waveform and the output Sum and Carry signals measured at 2 V (a) and 2.4 V (b).

**Supplementary Tables**

**Supplementary Table 1.** The summary of the key parameters of the nanogap Schottky diode is derived from the set of electrical measurements and different methods. The methods used for the extraction of each parameter are mentioned below the parameter.

| Ideality Factor  $n$ | | Donor Conc.  N_D_ (cm^-3^) | Barrier height  $\Phi_{B}$ (eV) | | | Series Resistance  R_S_ (kΩ) | |
| --- | --- | --- | --- | --- | --- | --- | --- |
| *I-V* | *Cheung* | *C-V* | *I-V* | *H(I) vs I* | *C-V* | *dV/dlnI vs I* | *H(I) vs I* |
| 1.75 | 1.86 | 1.13×10^18^ | 0.68 | 0.62 | 0.81 | 119 | 180 |

**Supplementary Table 2.** Summary of the notable emerging high-frequency Schottky diode technologies utilizing different materials, fabrication processes, and device structures from the literature. The comparison of these works is illustrated in Fig. 2h of the main manuscript.

| # | Material/Diode Structure | Deposition Method | *f_c_*_utoff_  (Intrinsic) | *I_on_/I_off_*  Ratio @1 V | *I_on_/I_off_*  Ratio @ indicated V | Year | Ref. |
| --- | --- | --- | --- | --- | --- | --- | --- |
| 1 | Al/ZnO/Au coplanar | Solution based (spin-coating) | 10.31 GHz | 4.4×10^5^ | 7.5×10^6^ at 2 V | 2023 | This work |
| 2 | Al/ZnO/Au & Al/IGZO/Ti-Pt | Solution based (spin-coating) | >100 GHz | ~10^4^ | ~10^5^ at  2V | 2022 | ^[10]^ |
| 3 | Al/Al-ZnO/Au  Coplanar | Solution based (spin coating) | 110 GHz | 9 × ${10}^{3}$ | 2 × ${10}^{6}$ at 3V | 2020 | ^[1]^ |
| 4 | Pt/p-WSe_2_/ITO (Vertical device on glass substrate) | Mechanical exfoliation and ALD process | 27 GHz | ~10^5^ |  | 2020 | ^[9]^ |
| 5 | Pd/MoS_2_/Au (Planar device on Kapton substrate) | Mechanical exfoliation and E-beam lithography | >10 GHz | ~10^2^ |  | 2019 | ^[8]^ |
| 6 | 2D Tellurium based | RF plasma and Solution | 1.3 GHz | 3×10^2^ |  | 2023 | ^[11]^ |
| 7 | In-Au/MoS_2_/In-Au  Coplanar | Mecanichal 2D-flake exfolation and transfer | 126 GHz | 6 × ${10}^{2}$ | 3.54 × ${10}^{3}$ at 1.5 V | 2021 | ^[12]^ |
| 8 | Pt/n-MoSe_2_/Graphene/Au  Vertical | Graphene wet-transfer in DI Water  MoSe2 Mechanical exfoliation and transfer | 200 GHz | 1 × ${10}^{4}$ | 1 × ${10}^{4}$ at 1V | 2023 | ^[13]^ |
| 9 | Ti-Pt/IGZO/Ti-Pt (Vertical device on acrylate subst.) | Sputtering (PVD process) | 1 GHz | 1.95×10^5^ | ­- | 2020 | ^[14]^ |
| 10 | Al/IGZO/Pt (Vertical device on glass and PET) | Sputtering (PVD process) | 16.7 GHz on glass, 6.3 GHz on PET | 9.2×10^4^ |  | 2015 | ^[7]^ |
| 11 | Al/IGZO/Cr-Au  Coplanar | IGZO deposited by Sputtering | 6.4 GHz | 7 × ${10}^{2}$ | 7 × ${10}^{3}$ at 2V | 2020 | ^[15]^ |

**Supplementary References**

[1] D. G. Georgiadou, J. Semple, A. A. Sagade, H. Forstén, P. Rantakari, Y.-H. Lin, F. Alkhalil, A. Seitkhan, K. Loganathan, H. Faber, T. D. Anthopoulos, *Nature Electronics* **2020**, 3, 718.

[2] P. Periasamy, H. L. Guthrey, A. I. Abdulagatov, P. F. Ndione, J. J. Berry, D. S. Ginley, S. M. George, P. A. Parilla, R. P. O'Hayre, *Advanced Materials* **2013**, 25, 1301.

[3] D. M. Pozar, *Microwave engineering*, Fourth edition. Hoboken, NJ : Wiley, [2012] ©2012, **2012**.

[4] J. Semple, S. Rossbauer, T. D. Anthopoulos, *ACS Applied Materials & Interfaces* **2016**, 8, 23167.

[5] J. Semple, D. G. Georgiadou, G. Wyatt-Moon, G. Gelinck, T. D. Anthopoulos, *Semiconductor Science and Technology* **2017**, 32, 123002.

[6] S. K. Cheung, N. W. Cheung, *Applied Physics Letters* **1986**, 49, 85.

[7] J. Zhang, Y. Li, B. Zhang, H. Wang, Q. Xin, A. Song, *Nature Communications* **2015**, 6, 7561.

[8] X. Zhang, J. Grajal, J. L. Vazquez-Roy, U. Radhakrishna, X. Wang, W. Chern, L. Zhou, Y. Lin, P.-C. Shen, X. Ji, X. Ling, A. Zubair, Y. Zhang, H. Wang, M. Dubey, J. Kong, M. Dresselhaus, T. Palacios, *Nature* **2019**, 566, 368.

[9] S. J. Yang, K.-T. Park, J. Im, S. Hong, Y. Lee, B.-W. Min, K. Kim, S. Im, *Nature Communications* **2020**, 11, 1574.

[10] K. Loganathan, H. Faber, E. Yengel, A. Seitkhan, A. Bakytbekov, E. Yarali, B. Adilbekova, A. AlBatati, Y. Lin, Z. Felemban, S. Yang, W. Li, D. G. Georgiadou, A. Shamim, E. Lidorikis, T. D. Anthopoulos, *Nature Communications* **2022**, 13, 3260.

[11] A. M. Askar, P. Palacios, F. Pasadas, M. Saeed, M. R. Mohammadzadeh, R. Negra, M. M. Adachi, *npj 2D Materials and Applications* **2023**, 7, 70.

[12] A. M. Askar, M. Saeed, A. Hamed, R. Negra, M. M. Adachi, *Nanoscale* **2021**, 13, 8940.

[13] S. Hong, C.-U. Hong, S. Lee, M. Jang, C. Jang, Y. Lee, L. J. Widiapradja, S. Park, K. Kim, Y.-W. Son, J.-G. Yook, S. Im, *Science Advances* **2023**, 9, eadh9770.

[14] E. Guerrero, A. Polednik, M. Ecker, A. Joshi-Imre, W. Choi, G. Gutierrez-Heredia, W. E. Voit, J. Maeng, *Advanced Electronic Materials* **2020**, 6, 1901210.

[15] G. Wyatt-Moon, K. M. Niang, C. B. Rider, A. J. Flewitt, *IEEE Electron Device Letters* **2020**, 41, 175.
